# Supplementary material for: Prolonged visual experience accelerates developmental synaptic downscaling via epigenetic regulation and Rab5c mediated AMPA receptor trafficking
Source: Commun Biol. 2026 Jan 9;9:230. doi: 10.1038/s42003-025-09507-5 (PMC12901309; doi:10.1038/s42003-025-09507-5)

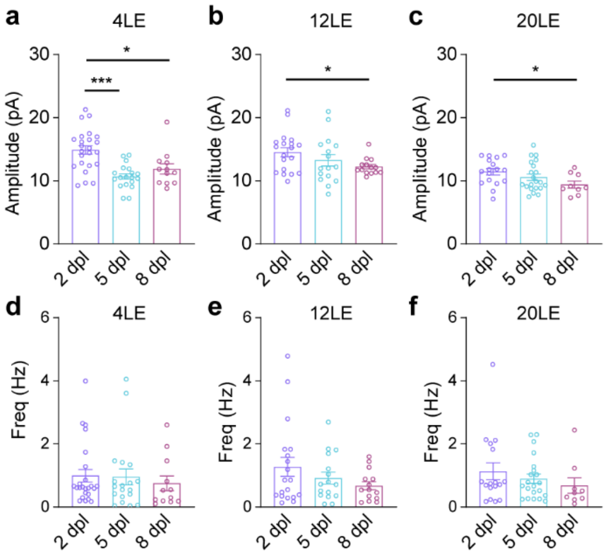

Supplementary Fig. 1 Developmental changes of mEPSC amplitude and frequency in tectal neurons under differential light-exposure conditions. **a**–**c** mEPSC amplitudes recorded at 4LE (**a**), 12LE (**b**), and 20LE (**c**) in neurons from tadpoles at 2, 5, and 8 dpl. **d**–**f** Corresponding mEPSC frequencies at 4LE (**d**), 12LE (**e**), and 20LE (**f**). 4LE: n =24, 19, 12; 12LE: n =19, 16, 14; 20LE: n = 17, 21, 9 for 2 dpl, 5 dpl, and 8 dpl. Statistical comparisons were performed using Brown-Forsythe and Welch ANOVA with Dunnett's T3 post-hoc tests: * $p < 0.05$, ** $p < 0.01$, *** $p < 0.001$.

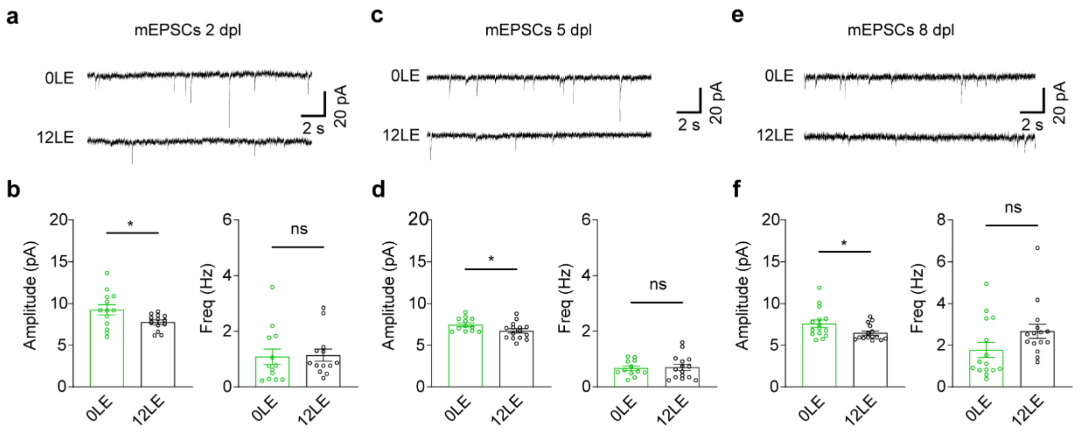

Supplementary Fig. 2 Visual deprivation (0LE) induces homeostatic upscaling of mEPSC amplitude in tectal neurons. **a**, **c**, **e** Representative whole-cell voltage-clamp traces of mEPSCs recorded from tectal neurons at 2 dpl (**a**), 5 dpl (**c**), and 8 dpl (**e**) following complete visual deprivation (0LE) versus standard 12 LE conditions. Scale bars: 20 pA, 2 s. **b**, **d**, **f** Quantification of mEPSC amplitudes at each time point reveals a significant increase under 0LE compared to 12LE (2 dpl: $n = 13, 13$; 5 dpl: $n = 12, 15$; 8 dpl: $n = 15, 15$; unpaired t-test, * $p < 0.05$). No significant differences in mEPSC frequency were observed between 0LE and 12LE groups at any time point.

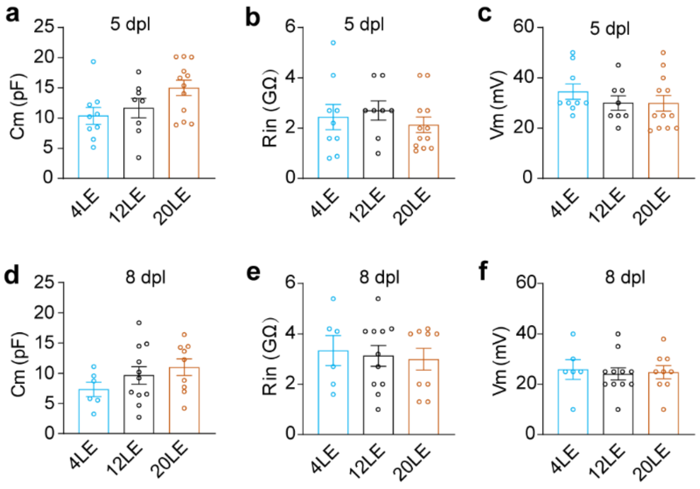

Supplementary Fig. 3 Intrinsic membrane properties of tectal neurons are unchanged by differential light exposure. **a**–**f** Membrane capacitance (Cm), input resistance (Rin) and resting membrane potential (Vm) are not significantly altered among 4LE-, 12LE-, and 20LE-treated neurons at 5 dpl (**a**–**c**) or 8 dpl (**d**–**f**). 5 dpl: *n* =9, 8, 12 for 4LE, 12LE and 20LE; 8 dpl: *n* =6, 11, 9 for 4LE, 12LE and 20LE.

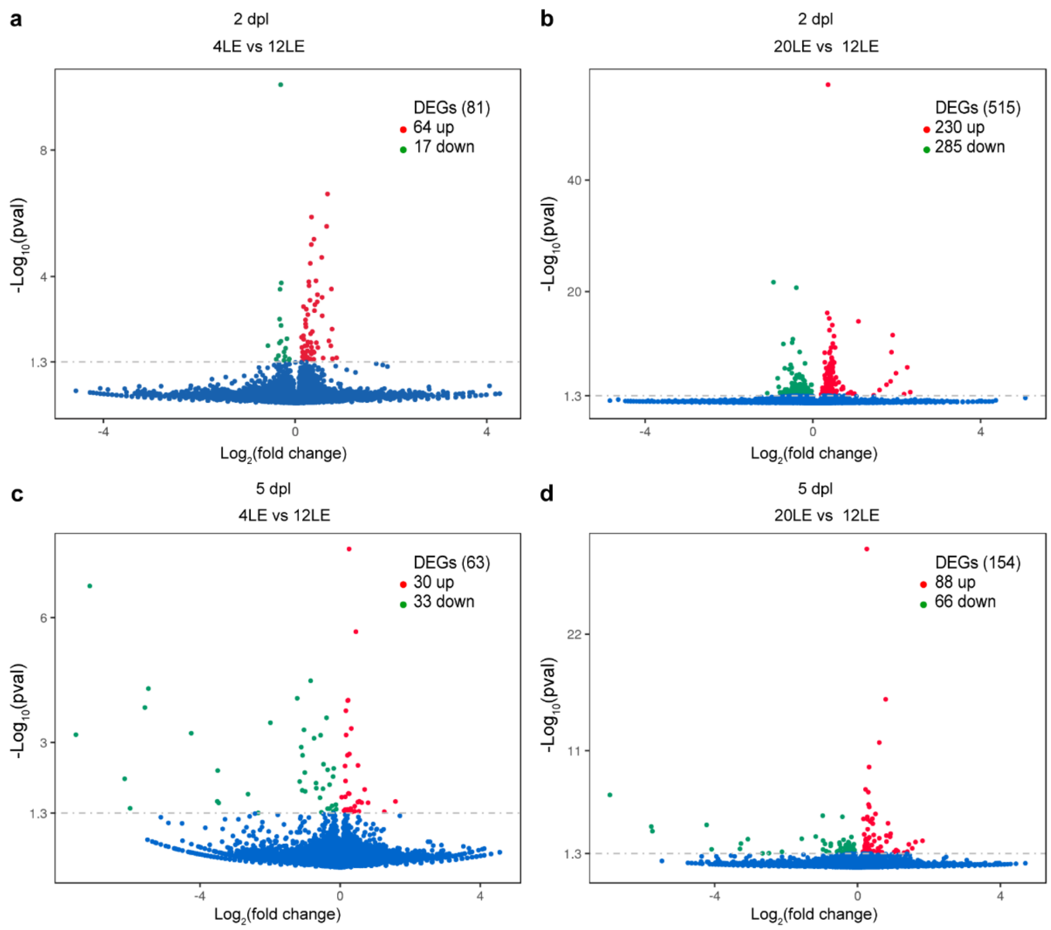

Supplementary Fig. 4 Volcano plot of all significantly regulated genes. **a** Differentially expressed genes in 4LE versus 12LE at 2 dpl. **b** Differentially expressed genes in 20LE versus 12LE at 2 dpl. **c** Differentially expressed genes in 4LE versus 12LE at 5 dpl. **d** Differentially expressed genes in 20LE versus 12LE at 5 dpl.

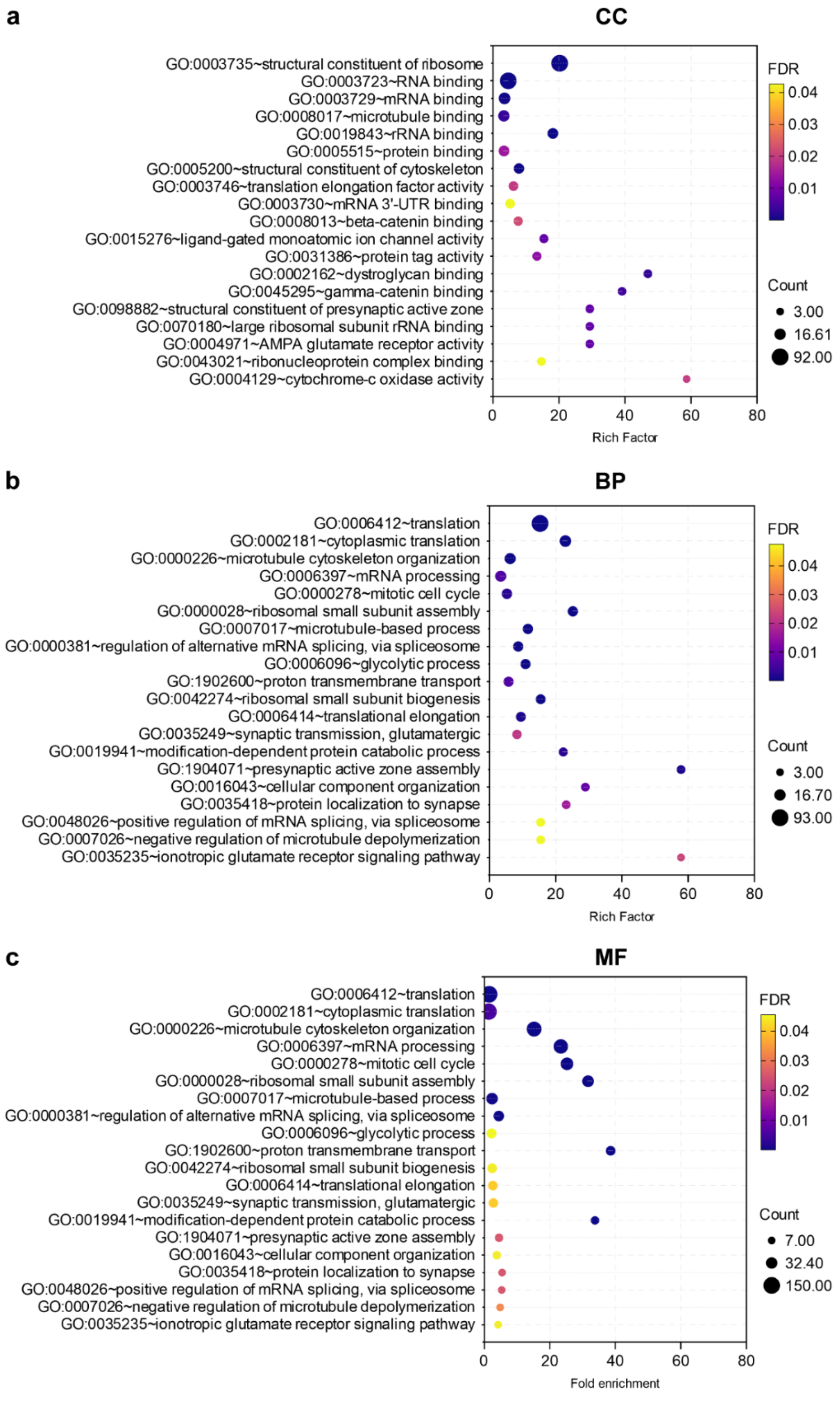

a
CC
GO:0003735~structural constituent of ribosome
GO:0003723~RNA binding
GO:0003729~mRNA binding
GO:0008017~microtubule binding
GO:0019843~rRNA binding
GO:0005515~protein binding
GO:0005200~structural constituent of cytoskeleton
GO:0003746~translation elongation factor activity
GO:0003730~mRNA 3'-UTR binding
GO:0008013~beta-catenin binding
GO:0015276~ligand-gated monoatomic ion channel activity
GO:0031386~protein tag activity
GO:0002162~dystroglycan binding
GO:0045295~gamma-catenin binding
GO:0098882~structural constituent of presynaptic active zone
GO:0070180~large ribosomal subunit rRNA binding
GO:0004971~AMPA glutamate receptor activity
GO:0043021~ribonucleoprotein complex binding
GO:0004129~cytochrome-c oxidase activity
Rich Factor
FDR
Count
3.00
16.61
92.00
b
BP
GO:0006412~translation
GO:0002181~cytoplasmic translation
GO:0000226~microtubule cytoskeleton organization
GO:0006397~mRNA processing
GO:0000278~mitotic cell cycle
GO:0000028~ribosomal small subunit assembly
GO:0007017~microtubule-based process
GO:0000381~regulation of alternative mRNA splicing, via spliceosome
GO:0006096~glycolytic process
GO:1902600~proton transmembrane transport
GO:0042274~ribosomal small subunit biogenesis
GO:0006414~translational elongation
GO:0035249~synaptic transmission, glutamatergic
GO:0019941~modification-dependent protein catabolic process
GO:1904071~presynaptic active zone assembly
GO:0016043~cellular component organization
GO:0035418~protein localization to synapse
GO:0048026~positive regulation of mRNA splicing, via spliceosome
GO:0007026~negative regulation of microtubule depolymerization
GO:0035235~ionotropic glutamate receptor signaling pathway
Rich Factor
FDR
Count
3.00
16.70
93.00
c
MF
GO:0006412~translation
GO:0002181~cytoplasmic translation
GO:0000226~microtubule cytoskeleton organization
GO:0006397~mRNA processing
GO:0000278~mitotic cell cycle
GO:0000028~ribosomal small subunit assembly
GO:0007017~microtubule-based process
GO:0000381~regulation of alternative mRNA splicing, via spliceosome
GO:0006096~glycolytic process
GO:1902600~proton transmembrane transport
GO:0042274~ribosomal small subunit biogenesis
GO:0006414~translational elongation
GO:0035249~synaptic transmission, glutamatergic
GO:0019941~modification-dependent protein catabolic process
GO:1904071~presynaptic active zone assembly
GO:0016043~cellular component organization
GO:0035418~protein localization to synapse
GO:0048026~positive regulation of mRNA splicing, via spliceosome
GO:0007026~negative regulation of microtubule depolymerization
GO:0035235~ionotropic glutamate receptor signaling pathway
Fold enrichment
FDR
Count
7.00
32.40
150.00

Supplementary Fig. 5 GO terms associated with all significantly regulated genes. **a**–**c** Bubble plots showing the top enriched GO categories in Cellular Component (CC, **a**), Biological Process (BP, **b**), and Molecular Function (MF, **c**) in the 20 LE group compared to 12 LE controls at 2 dpl. GO terms were ranked by -log10 (*p* value) and annotated using the DAVID Bioinformatics Resources.

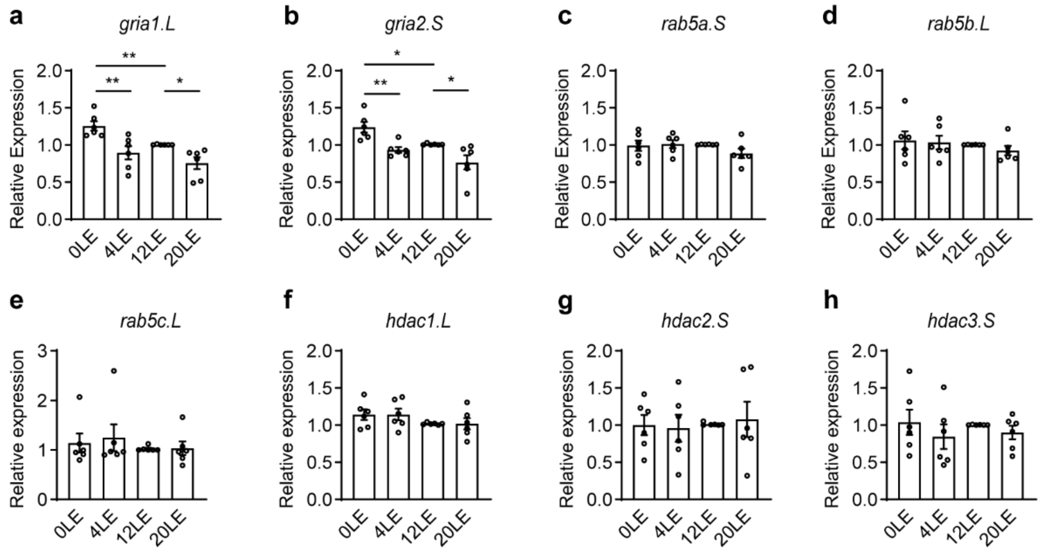

Supplementary Fig. 6 Quantitative RT-PCR analysis of mRNA expression at 2 dpl under different light-exposure conditions. **a–h** Relative mRNA levels of *gria1.L* (**a**), *gria2.S* (**b**), *rab5a.S* (**c**), *rab5b.L* (**d**), *rab5c.L* (**e**), *hdac1.L* (**f**), *hdac2.S* (**g**), and *hdac3.S* (**h**) were measured by qRT-PCR in optic-tectal tissue from tadpoles subjected to 0LE (0 hr light/24 hr dark), 4LE (4 hr light/20 hr dark), 12LE (12 hr light/12 hr dark), or 20LE (20 hr light/4 hr dark) at 2 dpl. Expression values were normalized to GAPDH and are presented as mean ± SEM ($n = 6$ per group). Statistical comparisons were made by one-way ANOVA with post hoc Newman-Keuls tests: * $p < 0.05$, ** $p < 0.01$.

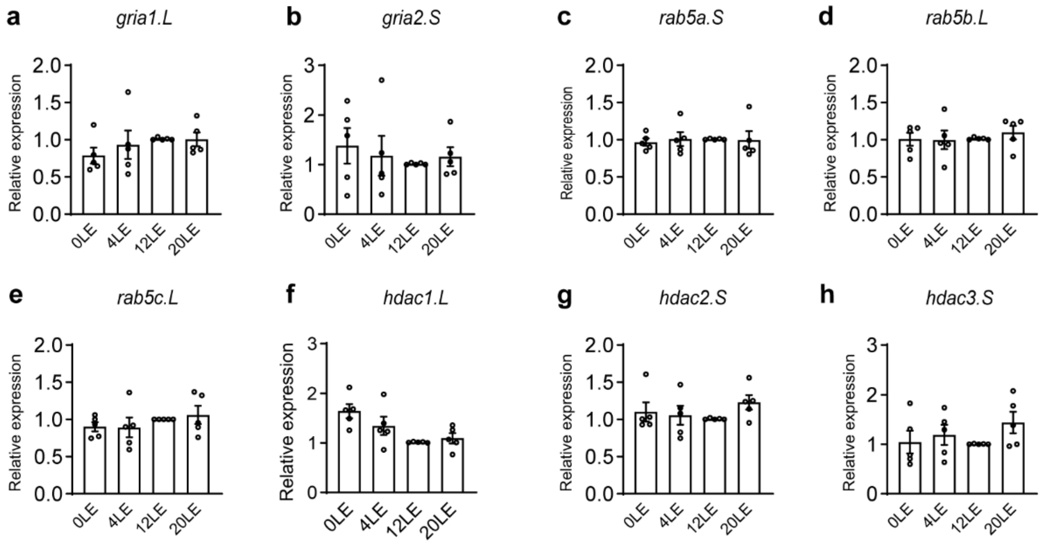

Supplementary Fig. 7 Quantitative RT-PCR analysis of mRNA expression at 5 dpl under different light-exposure conditions. **a**–**h** Relative mRNA levels of *gria1.L* (**a**), *gria2.S* (**b**), *rab5a.S* (**c**), *rab5b.L* (**d**), *rab5c.L* (**e**), *hdac1.L* (**f**), *hdac2.S* (**g**), and *hdac3.S* (**h**) were measured by qRT-PCR in optic-tectal tissue from tadpoles subjected to 0LE, 4LE, 12LE, or 20LE at 5 dpl. Expression values were normalized to GAPDH and are presented as mean ± SEM ($n = 5$ per group). Statistical comparisons were made by one-way ANOVA with post hoc Newman-Keuls tests: ** $p < 0.01$.

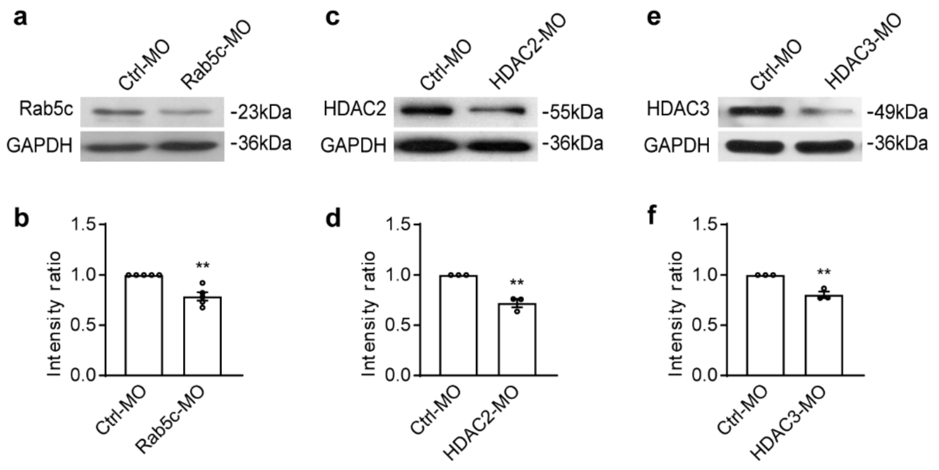

Supplementary Fig. 8 Validation of morpholino-mediated knockdown efficiency by Western blot. **a**–**b** Representative blot (**a**) and quantification (**b**) of Rab5c protein levels in Ctrl-MO versus Rab5c-MO-injected tectal lysates ($n = 5$ per group). **c**–**d** Representative blot (**c**) and quantification (**d**) of HDAC2 protein levels in Ctrl-MO versus HDAC2-MO-injected lysates ($n = 3$ per group). **e**–**f** Representative blot (**e**) and quantification (**f**) of HDAC3 protein levels in Ctrl-MO versus HDAC3-MO-injected lysates ($n = 3$ per group). Protein levels were normalized to GAPDH and are shown as mean ± SEM. ** $p < 0.01$ (unpaired t-test).

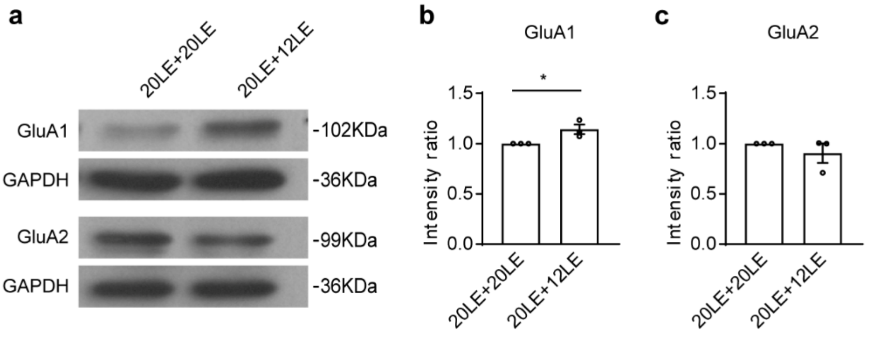

Supplementary Fig. 9 Recovery of GluA1 and GluA2 protein levels upon return to standard light conditions. **a** Representative Western blots of GluA1 and GluA2 in optic-tectal lysates from tadpoles continuously kept in 20LE (20LE + 20LE) for 4 days versus those returned to 12LE for 2 days after 20LE for 2 days (20LE + 12LE). **b**–**c** Quantification of GluA1 (**b**) and GluA2 (**c**) protein levels normalized to GAPDH ($n = 3$ per group). GluA1 level is significantly higher in the 20LE + 12 LE group compared with 20LE + 20LE (* $p < 0.05$, unpaired t-test).

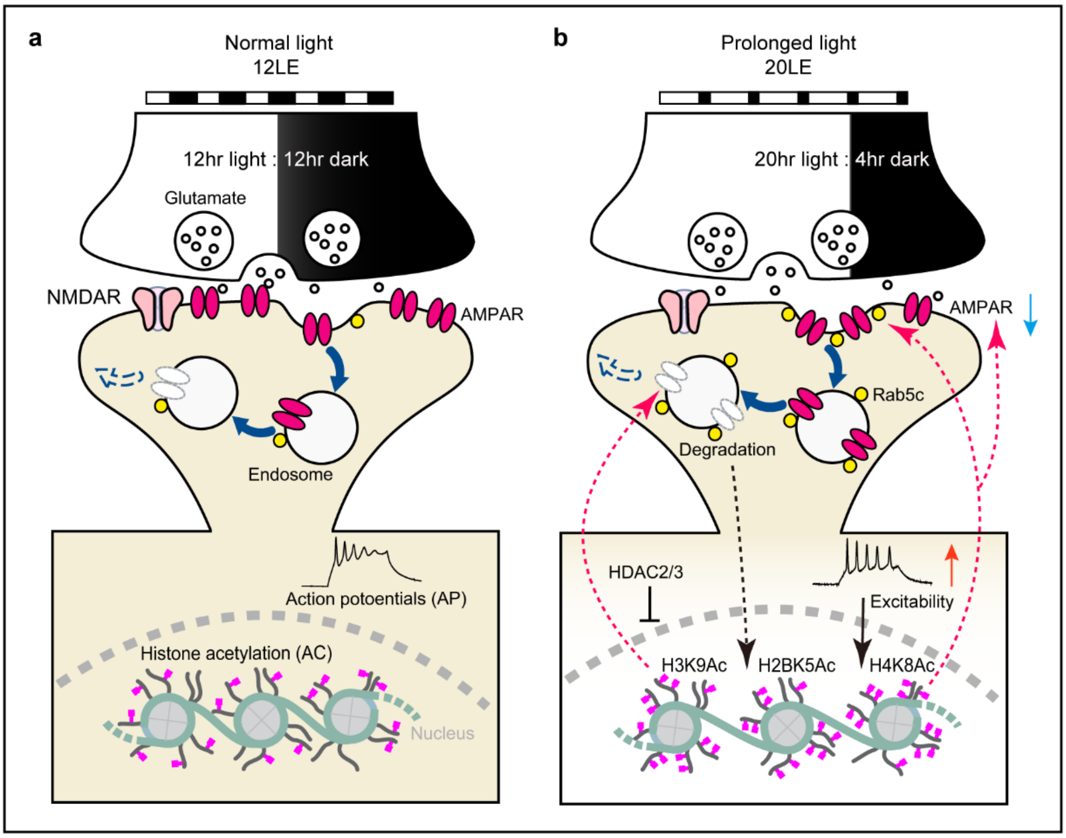

Supplementary Fig. 10 Schematic model of light exposure-induced synaptic downscaling. Comparison between normal light exposure (12LE, **a**) and prolonged light exposure (20LE, **b**) illustrating a coordinated homeostatic cascade. Prolonged visual experience induces activity-dependent epigenetic remodeling characterized by increased histone acetylation at H3K9, H2BK5, and H4K8 through HDAC2/3 modulation. These chromatin changes suppress *GluA1* and *GluA2* transcription, thereby reducing AMPAR synthesis. Concurrently, elevated synaptosomal Rab5c promotes AMPAR endocytosis, consolidating the reduction of synaptic GluA2 and sustaining decreased excitatory drive. HDAC2 primarily regulates AMPAR subunit transcription via chromatin remodeling, while HDAC3 additionally modulates Rab5c protein abundance, establishing a mechanistic link between epigenetic regulation and receptor trafficking.

Supplementary Fig. 11 All Western blot images presented in the manuscript.

Fig. 5a GluA1 and GluA2

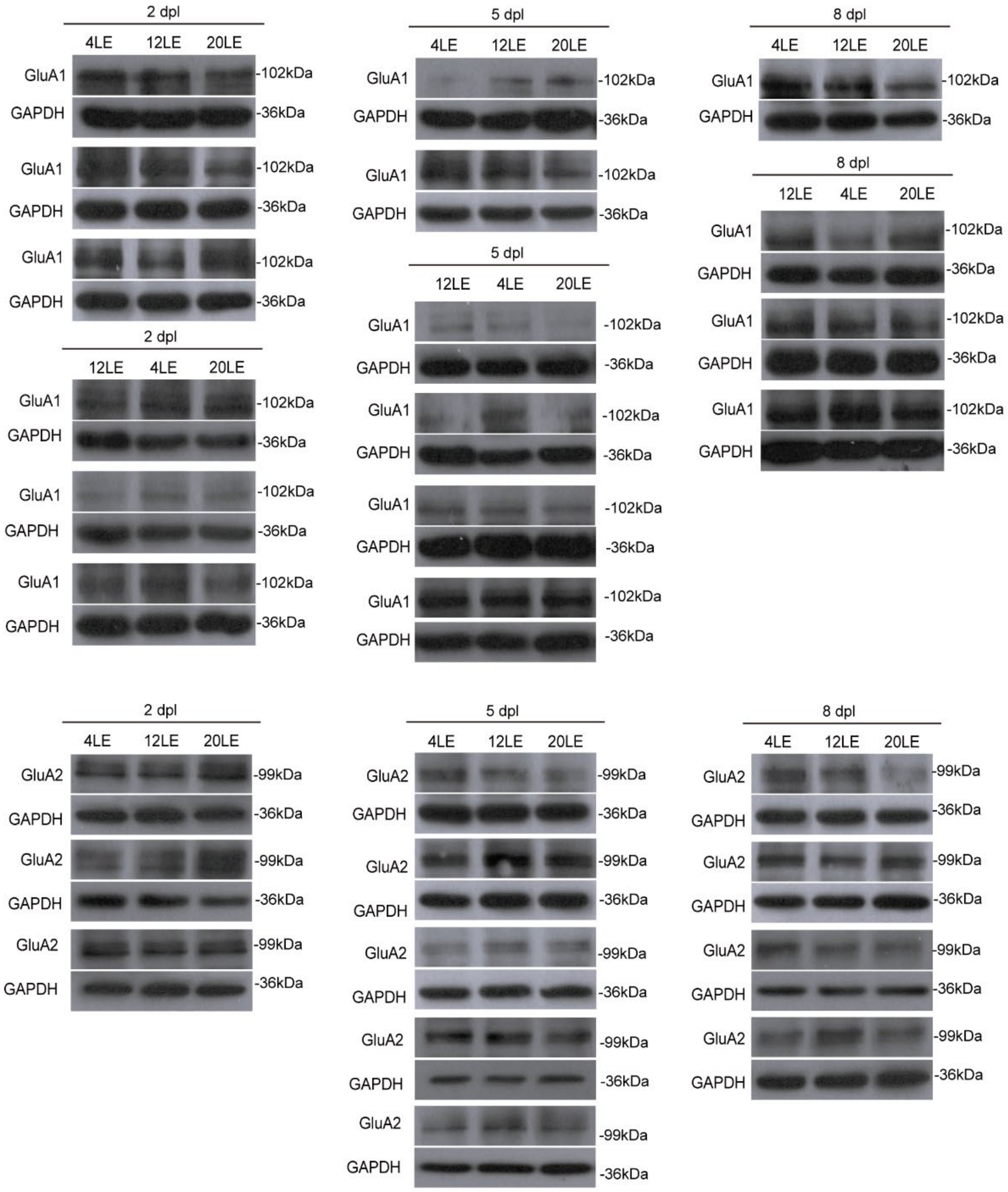

Fig. 5a Rab5c

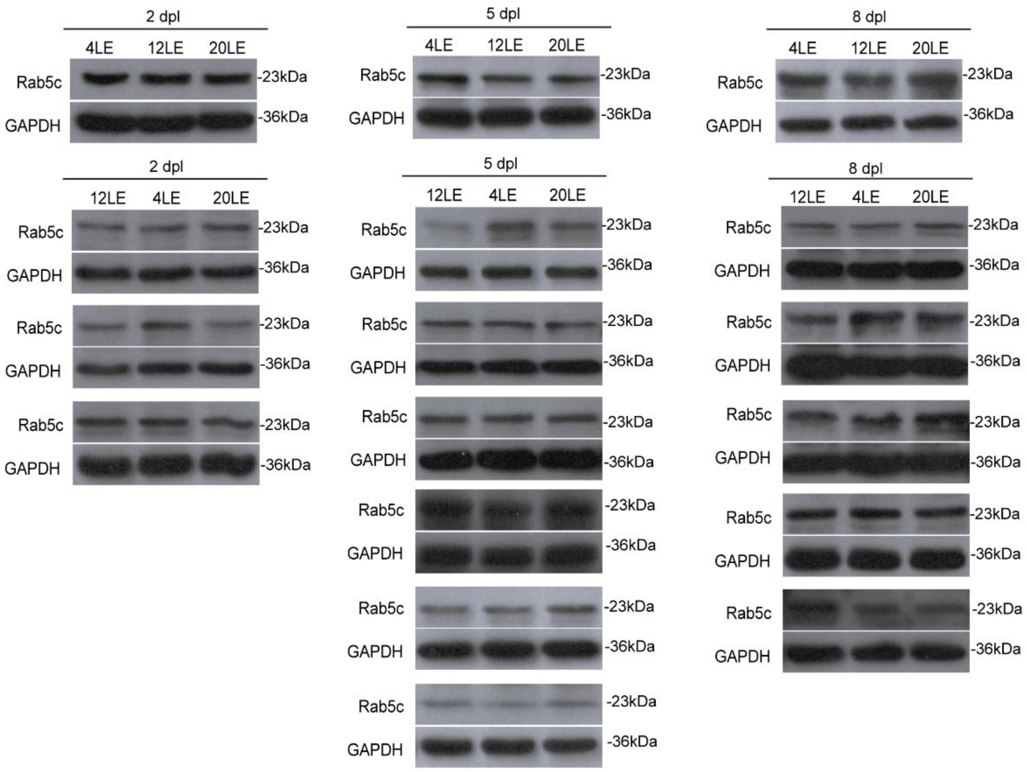

Fig. 5c

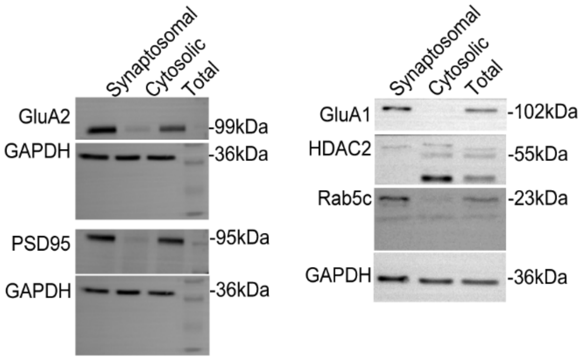

Fig. 5d

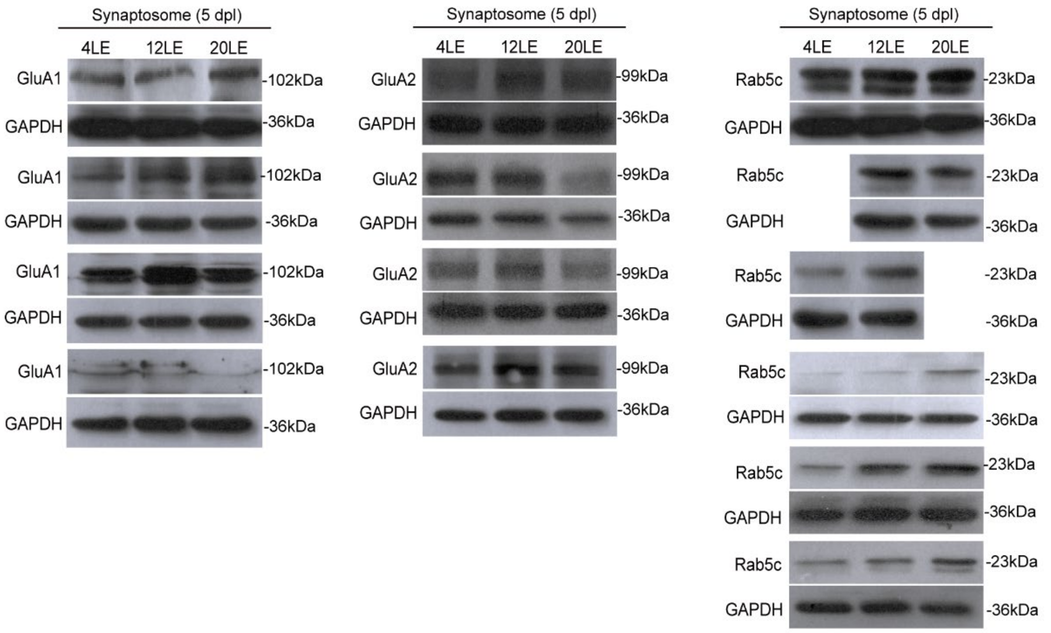
Synaptosome (5 dpl)
4LE
12LE
20LE
GluA1
GAPDH
-102kDa
-36kDa
GluA2
-99kDa
Rab5c
-23kDa

Fig. 7g

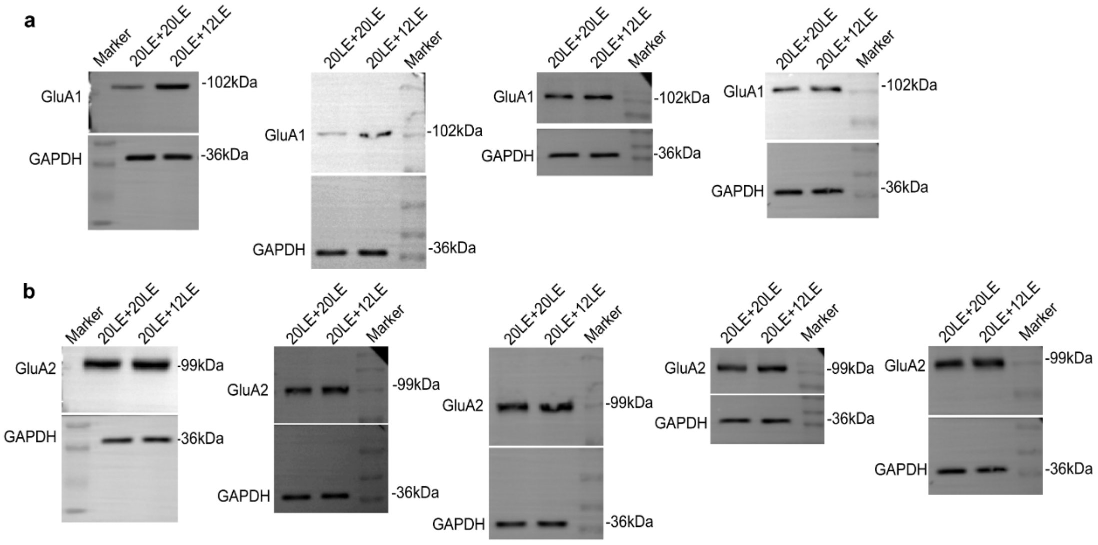
a
b
Marker
20LE+20LE
20LE+12LE
GluA1
GluA2
GAPDH
-102kDa
-99kDa
-36kDa

Fig. 8a H3K9Ac

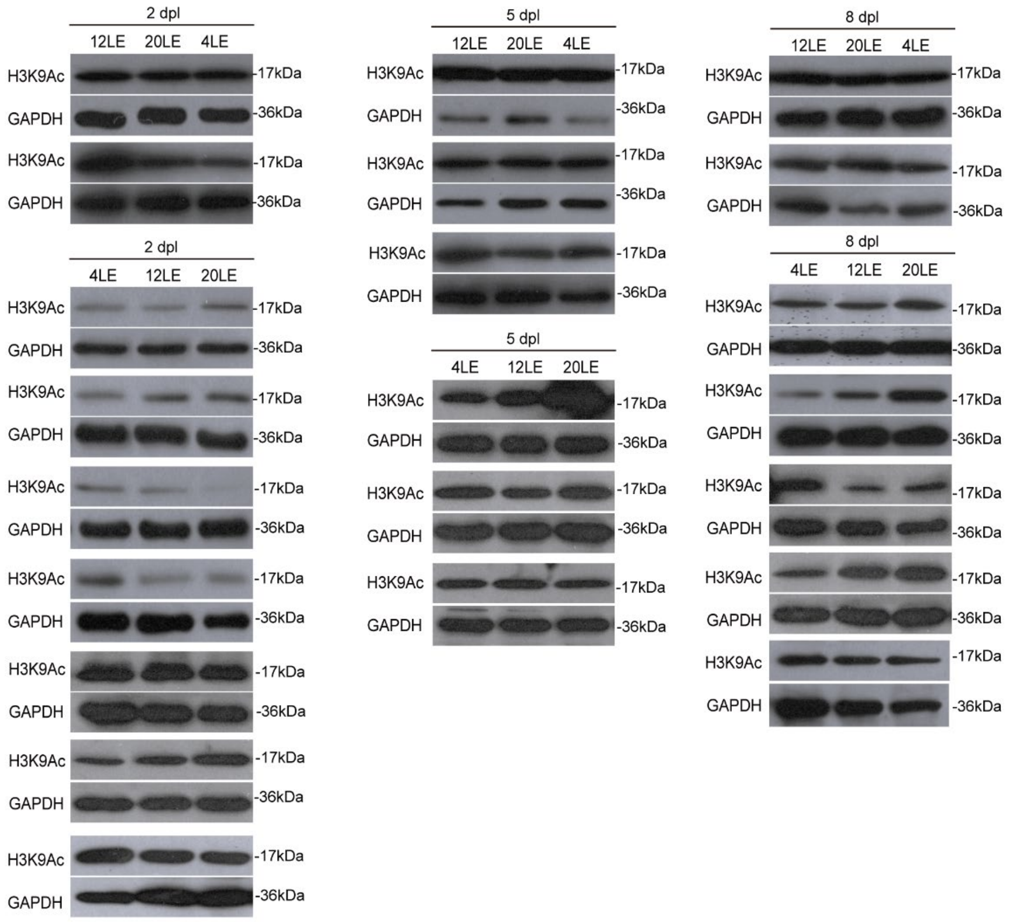

Fig. 8a H2BK5Ac

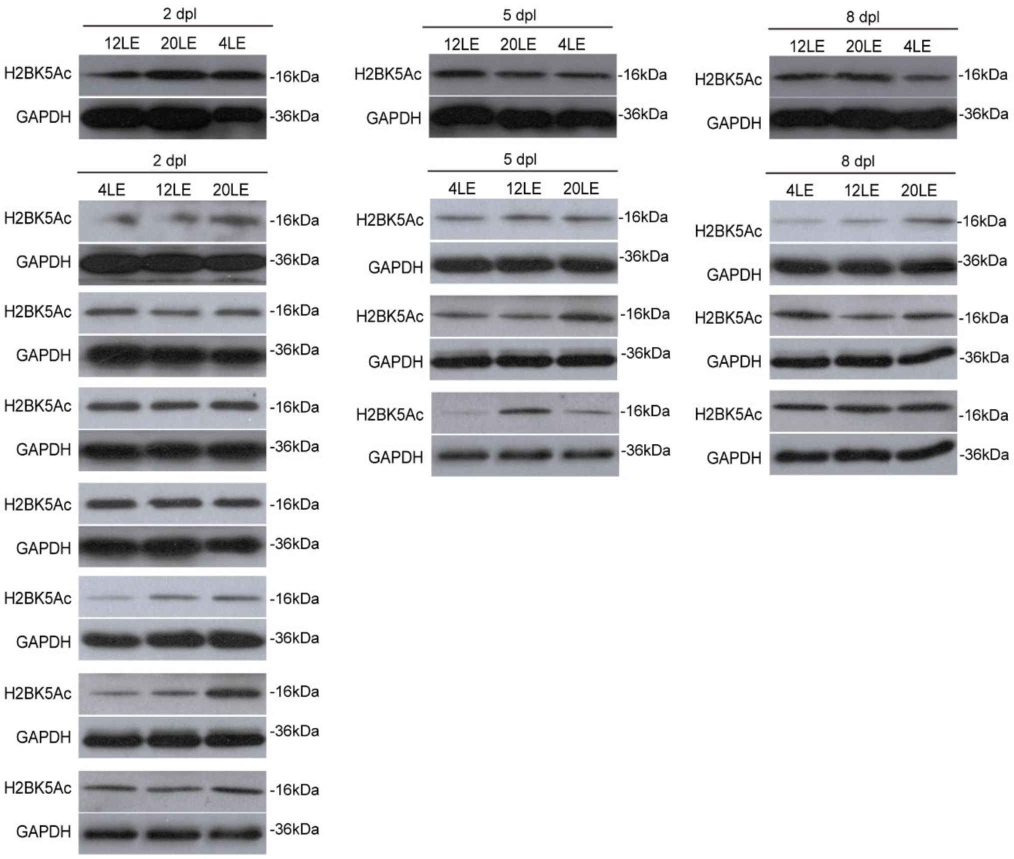

Fig. 8a H4K8Ac

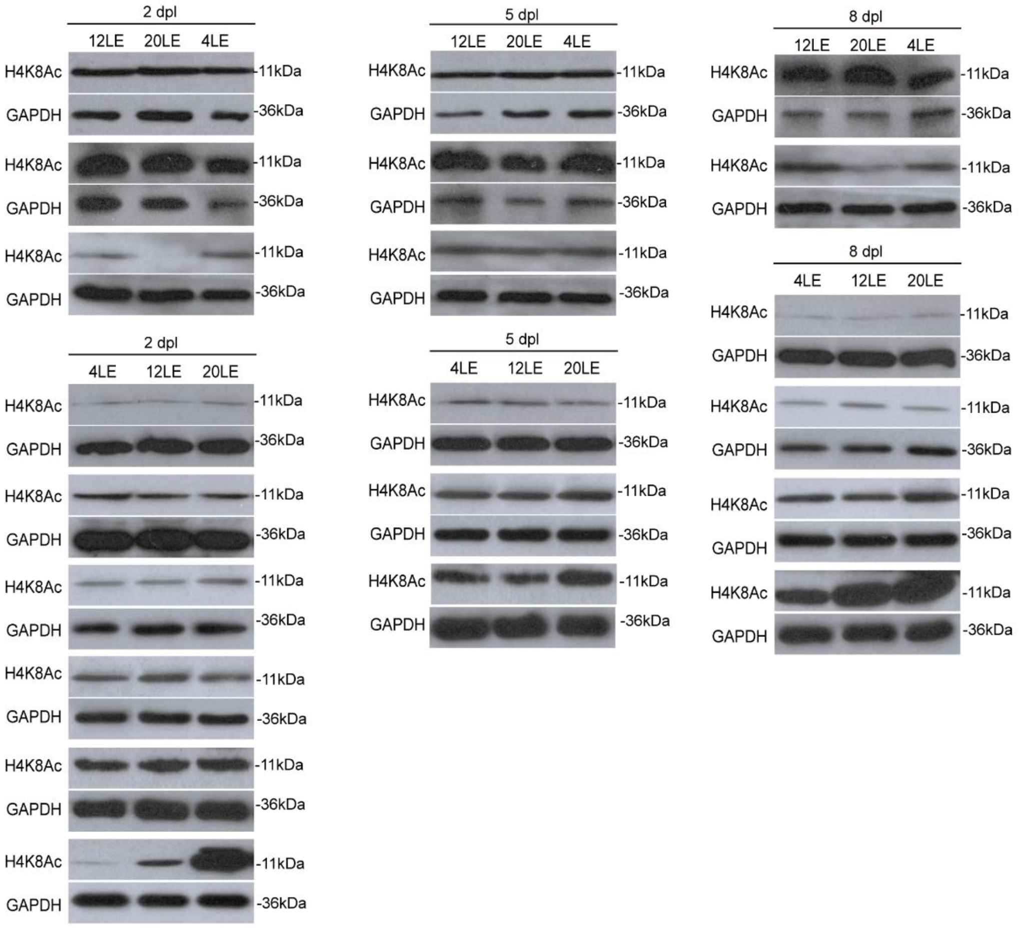

Fig. 9g

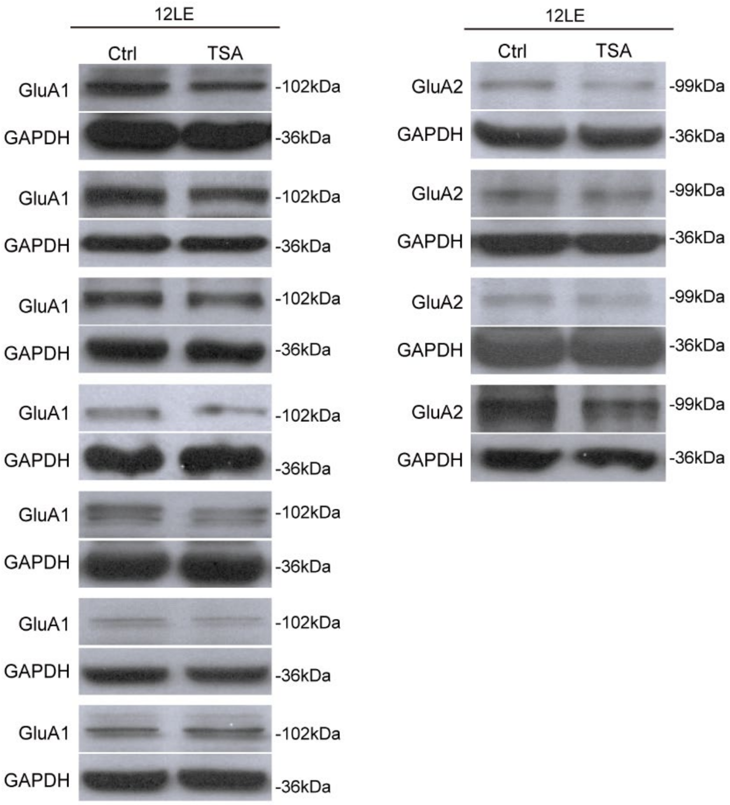
12LE
Ctrl
TSA
GluA1
GAPDH
-102kDa
-36kDa
GluA2
-99kDa

Fig. 9j

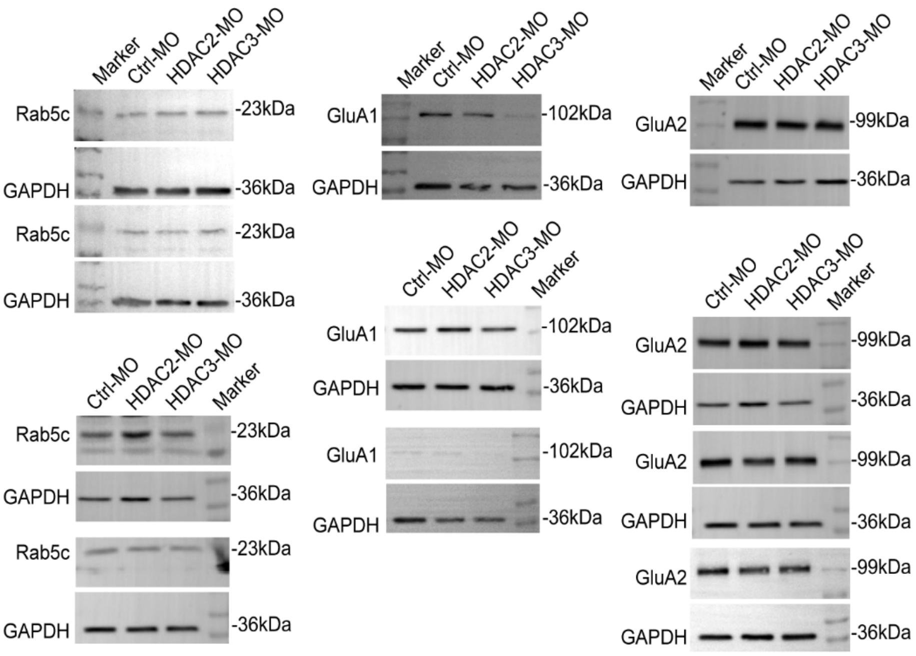
Marker
Ctrl-MO
HDAC2-MO
HDAC3-MO
Rab5c
GAPDH
GluA1
GluA2
-23kDa
-36kDa
-102kDa
-99kDa

Fig. 9n

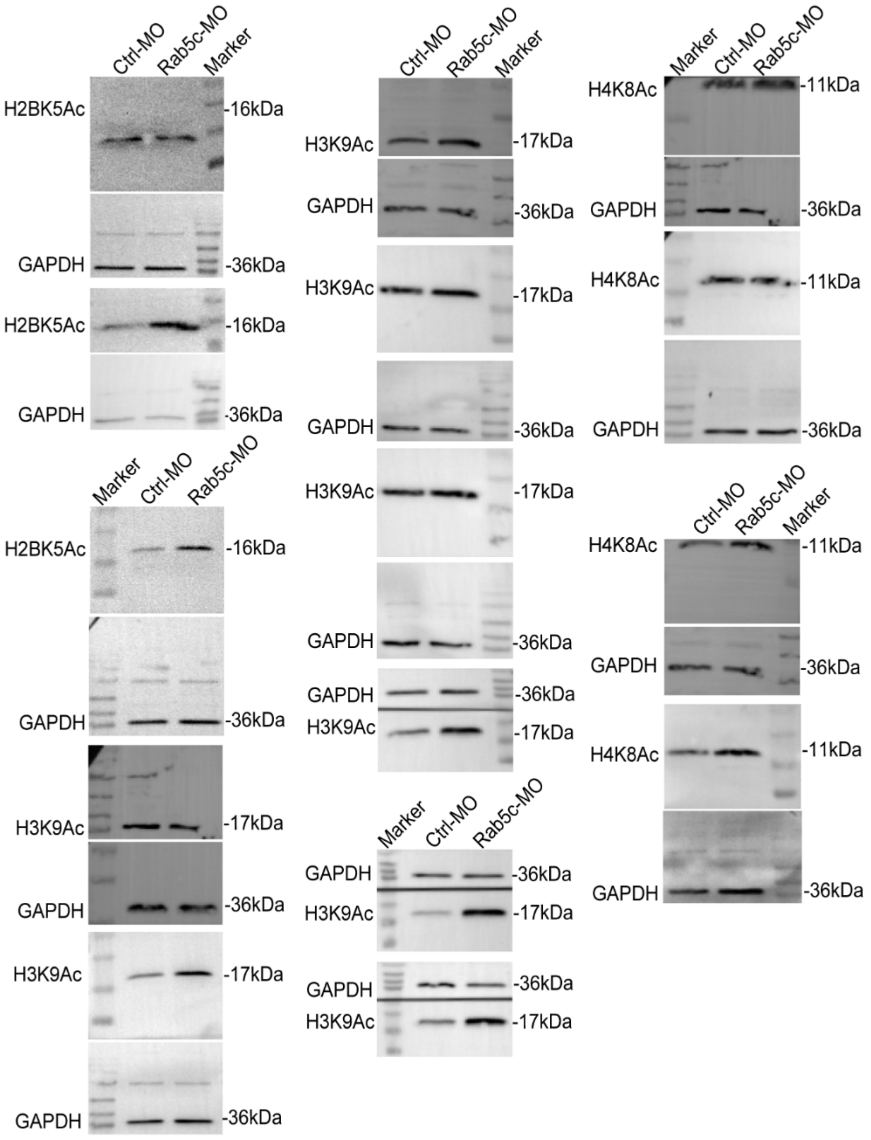
Ctrl-MO
Rab5c-MO
Marker
H2BK5Ac
-16kDa
GAPDH
-36kDa
H3K9Ac
-17kDa
H4K8Ac
-11kDa

Supplementary Fig. 9a

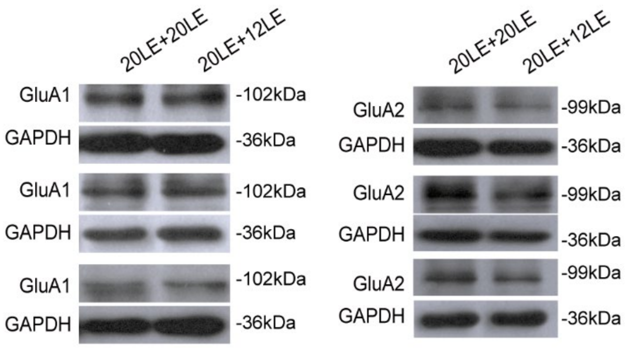

Supplement: Supplementary file 1 — Supplementary Information [file 42003_2025_9507_MOESM1_ESM.pdf]
