# Supplementary material for: Prolonged visual experience accelerates developmental synaptic downscaling via epigenetic regulation and Rab5c mediated AMPA receptor trafficking
Source: Commun Biol. 2026 Jan 9;9:230. doi: 10.1038/s42003-025-09507-5 (PMC12901309; doi:10.1038/s42003-025-09507-5)
Supplement: Supplementary file 2 — Description of Additional Supplementary Files [file 42003_2025_9507_MOESM2_ESM.docx]

Description of Additional Supplementary File

File name: Supplementary Data 1

Description: Source data for all graphs can be found in the Supplementary Data
