# Supplementary material for: Prolonged visual experience accelerates developmental synaptic downscaling via epigenetic regulation and Rab5c mediated AMPA receptor trafficking
Source: Commun Biol. 2026 Jan 9;9:230. doi: 10.1038/s42003-025-09507-5 (PMC12901309; doi:10.1038/s42003-025-09507-5)
Supplement: Supplementary file 4 — Reporting Summary [file 42003_2025_9507_MOESM4_ESM.pdf]

nature portfolio

Corresponding author(s): Wanhua Shen
Last updated by author(s): Dec 7, 2025

Reporting Summary

Nature Portfolio wishes to improve the reproducibility of the work that we publish. This form provides structure for consistency and transparency in reporting. For further information on Nature Portfolio policies, see our Editorial Policies and the Editorial Policy Checklist.

Statistics

For all statistical analyses, confirm that the following items are present in the figure legend, table legend, main text, or Methods section.

n/a	Confirmed	
☐	☒	The exact sample size (*n*) for each experimental group/condition, given as a discrete number and unit of measurement
☐	☒	A statement on whether measurements were taken from distinct samples or whether the same sample was measured repeatedly
☐	☒	The statistical test(s) used AND whether they are one- or two-sided<br>*Only common tests should be described solely by name; describe more complex techniques in the Methods section.*
☐	☒	A description of all covariates tested
☐	☒	A description of any assumptions or corrections, such as tests of normality and adjustment for multiple comparisons
☐	☒	A full description of the statistical parameters including central tendency (e.g. means) or other basic estimates (e.g. regression coefficient) AND variation (e.g. standard deviation) or associated estimates of uncertainty (e.g. confidence intervals)
☐	☒	For null hypothesis testing, the test statistic (e.g. *F*, *t*, *r*) with confidence intervals, effect sizes, degrees of freedom and *P* value noted<br>*Give P values as exact values whenever suitable.*
☒	☐	For Bayesian analysis, information on the choice of priors and Markov chain Monte Carlo settings
☒	☐	For hierarchical and complex designs, identification of the appropriate level for tests and full reporting of outcomes
☒	☐	Estimates of effect sizes (e.g. Cohen's *d*, Pearson's *r*), indicating how they were calculated

Our web collection on statistics for biologists contains articles on many of the points above.

Software and code

Policy information about availability of computer code

Data collection	Nikon and Zeiss Zen software (2012) were used to acquire images. Imaris 7.4.2 was used to reconstruct and quantify morphological data. Multiclamp 700A/ClampFit 10 was used to collect/analyze electrophysiological data.
Data analysis	MiniAnalysis Program (Synaptosoft) and ClampFit 10 were used to analyze recording data. Microsoft office 2019, Adobe illustrator 2024, Adobe photoshop 2024 and GraphPad Prim 10 were used to organize pictures and analyze data. Differentially expressed genes (DEGs) were identified using DESeq (version 1.12.0), and gene expression was assessed using Cufflinks (version 2.1.1). Functional annotation analysis was conducted using DAVID (https://david.ncifcrf.gov).

For manuscripts utilizing custom algorithms or software that are central to the research but not yet described in published literature, software must be made available to editors and reviewers. We strongly encourage code deposition in a community repository (e.g. GitHub). See the Nature Portfolio guidelines for submitting code & software for further information.

Data

Policy information about availability of data

All manuscripts must include a data availability statement. This statement should provide the following information, where applicable:

- Accession codes, unique identifiers, or web links for publicly available datasets
- A description of any restrictions on data availability
- For clinical datasets or third party data, please ensure that the statement adheres to our policy

All raw and processed data in this study will be made available upon request.

Research involving human participants, their data, or biological material

Policy information about studies with human participants or human data. See also policy information about sex, gender (identity/presentation), and sexual orientation and race, ethnicity and racism.

Reporting on sex and gender	Does not apply.
Reporting on race, ethnicity, or other socially relevant groupings	Does not apply.
Population characteristics	Does not apply.
Recruitment	Does not apply.
Ethics oversight	Does not apply.

Note that full information on the approval of the study protocol must also be provided in the manuscript.

Field-specific reporting

Please select the one below that is the best fit for your research. If you are not sure, read the appropriate sections before making your selection.

☒ Life sciences ☐ Behavioural & social sciences ☐ Ecological, evolutionary & environmental sciences

For a reference copy of the document with all sections, see nature.com/documents/nr-reporting-summary-flat.pdf

Life sciences study design

All studies must disclose on these points even when the disclosure is negative.

Sample size	Statistical test details and sample sizes were shown in figure legends and methods. No sample size calculation was performed. The sample sizes for recordings, Western blots were estimated from our previously published experiments.
Data exclusions	Data were not excluded from analysis.
Replication	For all data collections, at least three independent biological replicates were used for all statistical analyses.
Randomization	Batches of animals were randomly selected from experimental groups. Multiple batches of animals were used. Transfected tadpoles were screened and single neurons in the optic tectum were randomly selected for recordings and imaging.
Blinding	Groups for data of electrophysiological recordings were blindly performed by Lijun Zheng and analyzed by Wanhua Shen.

Reporting for specific materials, systems and methods

We require information from authors about some types of materials, experimental systems and methods used in many studies. Here, indicate whether each material, system or method listed is relevant to your study. If you are not sure if a list item applies to your research, read the appropriate section before selecting a response.

Materials & experimental systems

n/a	Involved in the study
☐	☒ Antibodies
☒	☐ Eukaryotic cell lines
☒	☐ Palaeontology and archaeology
☐	☒ Animals and other organisms
☒	☐ Clinical data
☒	☐ Dual use research of concern
☒	☐ Plants

Methods

n/a	Involved in the study
☒	☐ ChIP-seq
☒	☐ Flow cytometry
☒	☐ MRI-based neuroimaging

Antibodies

Antibodies used

anti-GAPDH (1:30000, ABS16, Millipore), anti-Rab5c (1:2000, ABP55966, Abbkine), anti-GluA1 (1:2000, TA332438S, Origene), anti-GluA2 (1:1000, pps050, R&D systems), anti-HDAC2 (1:1000, ab137364, Abcam), anti-HDAC3 (1:1000, ab16047, Abcam), anti-H3K9Ac (1:2000, ab10812, Abcam), anti-H2BK5Ac (1:2000, ab408867, Abcam), anti-H4K8Ac (1:1000, ab45166, Abcam),horseradish peroxidase (HRP)-conjugated secondary antibodies (1:2000, CWBIO), anti-PSD95 (1:2000, ab2723, Abcam)

Validation

Antibodies of anti-GAPDH, anti-GluA1, anti-GluA2,anti-HDAC2, anti-HDAC3, anti-H3K9Ac anti-H2BK5Ac, and anti-H4K8Ac were validated in our previously published articles (Gao, J et al, 2016; Ruan, H et al, 2017; Liao, Y, et al, 2020; Gao, J et al, 2022).

Animals and other research organisms

Policy information about studies involving animals; ARRIVE guidelines recommended for reporting animal research, and Sex and Gender in Research

Laboratory animals

Adult Xenopus laevis (male and female) were originally purchased from Nasco and raised in house for mating to produce tadpoles.

Wild animals

No wild animals were used in this study.

Reporting on sex

Sex determination was not required for the tadpoles used in this study.

Field-collected samples

No field-collected samples were used in this study.

Ethics oversight

The Xenopus work was performed under a study protocol as approved by the ethics committee of Hangzhou Normal University.

Note that full information on the approval of the study protocol must also be provided in the manuscript.

Plants

Seed stocks

Does not apply.

Novel plant genotypes

Does not apply.

Authentication

Does not apply.
